# Identification of key genes and immune mechanisms in atrial fibrillation: An observational bioinformatics and single-cell transcriptome study

**DOI:** 10.1097/MD.0000000000048505

**Published:** 2026-04-24

**Authors:** Yilin Su, Zhijie Ye, Junliang Hou, Xiaolan Lin, Chongkun Zheng, Laiying Xu, Yan Zhang

**Affiliations:** a Affiliated Fuzhou First Hospital of Fujian Medical University, No. 190, Dadao Road, Fuzhou, Fujian, China.

**Keywords:** atrial fibrillation, bioinformatics, immune infiltration, LASSO regression, observational study, single-cell RNA sequencing, therapeutic targets

## Abstract

Atrial fibrillation (AF) is a common arrhythmia associated with immune dysregulation and complex molecular mechanisms. This observational bioinformatics study aimed to identify key genes involved in AF, explore their functional roles, and uncover potential therapeutic targets. Public gene expression datasets (GSE79768 and GSE41177) and single-cell ribonucleic acid sequencing datasets (GSE224995 and GSE261170) were analyzed. Differentially expressed genes were screened using the limma package, and enrichment analyses (Gene Ontology/Kyoto Encyclopedia of Genes and Genomes) were conducted. Key genes were selected via Least Absolute Shrinkage and Selection Operator regression and validated in external datasets. Immune cell infiltration was assessed using the CIBERSORT algorithm, while single-cell ribonucleic acid sequencing analysis provided cell-type-specific expression and cell-cell interaction insights. We identified 85 differentially expressed genes, among which LBH, C8orf4, INPP5A, CHGB, and B3GALTL showed consistent differential expression and diagnostic performance (area under the curve ≥ 0.70). Immune analysis revealed a pro-inflammatory microenvironment in AF, characterized by elevated neutrophils and classically activated macrophages and reduced alternatively activated macrophages and Tregs. Single-cell analysis confirmed the cell-specific expression of key genes and highlighted intercellular communication between immune and stromal cells. Our findings suggest that immune imbalance and mitochondrial dysfunction are central to AF pathogenesis. The identified genes may serve as promising diagnostic biomarkers and therapeutic targets for AF.

## 1. Introduction

Atrial fibrillation (AF) is the most common sustained cardiac arrhythmia worldwide, affecting more than 33 million people, with its prevalence expected to rise significantly in the coming decades.^[1,2]^ AF is a progressive disease that begins as paroxysmal events and, if untreated, can develop into persistent and permanent forms.^[3,4]^ It is a major contributor to stroke, heart failure, and increased mortality, placing a substantial burden on healthcare systems.^[5,6]^ The incidence of AF increases with age and is commonly associated with risk factors such as hypertension, coronary artery disease, diabetes mellitus, and heart failure, all of which contribute to atrial remodeling and electrical instability.^[7,8]^

Despite its high prevalence, the molecular mechanisms driving AF remain poorly understood, limiting the development of effective therapeutic strategies.^[9]^ Current treatments focus primarily on symptom management and stroke prevention, including rate/rhythm control medications (e.g., β-blockers, amiodarone) and direct oral anticoagulants.^[10,11]^ However, these approaches have limited efficacy and potential adverse effects, underscoring the need for a deeper understanding of AF pathophysiology to develop targeted therapies.^[11,12]^

Atrial remodeling, characterized by electrical, structural, and metabolic alterations, plays a critical role in AF progression.^[13–15]^ Recent studies have highlighted the involvement of immune dysregulation, chronic inflammation, and mitochondrial dysfunction in AF pathogenesis.^[16–18]^ Dysregulated gene expression in immune cells, oxidative stress pathways, and atrial extracellular matrix components has been implicated in the disease.^[19–21]^ However, the precise molecular mechanisms and key regulatory genes remain unclear.

With the advancement of bioinformatics, transcriptomic and single-cell analyses have provided new insights into disease-associated gene expression and cellular interactions.^[22,23]^ Here, using bulk ribonucleic acid-sequencing (RNA-seq) and single-cell RNA sequencing data (scRNA-seq), we systematically analyzed AF-associated gene expression, immune cell infiltration, and intercellular communication. We identified key differentially expressed genes (DEGs) in AF and performed functional enrichment analysis to explore their biological roles. Machine learning (least absolute shrinkage and selection operator [LASSO] regression) was applied to screen for potential diagnostic markers, followed by validation in independent datasets. Additionally, we investigated immune cell interactions and AF-specific immune alterations using CIBERSORT.

By integrating transcriptomic and single-cell analyses, this study aims to provide a comprehensive molecular landscape of AF, uncover potential biomarkers for AF diagnosis and stratification, and identify new therapeutic targets for precision medicine approaches.

## 2. Materials and methods

### 2.1. Ethical approval

This study was conducted using publicly available datasets (GSE79768, GSE41177, GSE224995, and GSE261170) from the Gene Expression Omnibus database. Therefore, ethical approval and informed consent were not required.

### 2.2. Data acquisition

Publicly available gene expression datasets were obtained from the Gene Expression Omnibus database. The bulk RNA sequencing dataset GSE79768, which includes atrial tissue samples from patients with AF and healthy controls, was used as the primary dataset for DEG screening. The dataset GSE41177 was used for independent validation.

For scRNA-seq analysis, GSE224995 (healthy individuals) and GSE261170 (AF patients) were retrieved to investigate cell-type-specific expression and intercellular heterogeneity. All raw data (series matrix and count files) were downloaded and preprocessed in R, using appropriate normalization and quality control pipelines.

### 2.3. Identification of DEGs

The Kyoto Encyclopedia of Genes and Genomes (KEGG) gene set (c2.cp.kegg.v11.0.symbols) was downloaded from the molecular signatures database. The R package psych (version 2.5.6; William Revelle from Northwestern University, Evanston) was used to perform Spearman correlation analysis between each biomarker and all other genes across all samples in GSE79768, obtaining correlation coefficients. Genes were then ranked based on these correlation coefficients to generate a list of correlated genes for each biomarker. Using these ranked lists, Gene Set Enrichment Analysis (GSEA) was performed with the R package clusterProfiler (version 4.12.6; Guangzhou Institutes of Biomedicine and Health, Chinese Academy of Sciences, Guangzhou, China), with the thresholds set as adj.*P* value < .05 and |NES| > 1, against the KEGG pathways. Finally, the top 5 most significant pathways were visualized using the R package enrichplot (version 1.24.4; Guangzhou Institutes of Biomedicine and Health, Chinese Academy of Sciences, Guangzhou, China).

### 2.4. Functional enrichment analysis

To investigate the biological significance of the identified DEGs, Gene Ontology (GO) and KEGG pathway enrichment analyses were conducted using the clusterProfiler package. GO enrichment analysis categorized genes into 3 functional domains: biological processes, cellular components, and molecular functions. KEGG pathway analysis was performed to identify significantly enriched signaling pathways associated with AF. Enrichment results were visualized using bar plots and bubble plots to highlight the top 10 most significantly enriched GO terms and KEGG pathways (*P* < .05).

### 2.5. Identification of key genes via machine learning

To identify key AF-associated genes with potential diagnostic value, LASSO regression was applied using the glmnet package in R. The model was trained on the GSE79768 dataset, and the optimal gene set was selected based on the minimum lambda value. The identified genes were then validated in an independent dataset (GSE41177) to assess their robustness. To evaluate the diagnostic performance of these key genes, receiver operating characteristic (ROC) curve analysis was performed using the pROC package, and the area under the curve (AUC) values were calculated. Genes with AUC ≥ 0.7 were considered to have strong predictive power for distinguishing AF patients from healthy individuals.

### 2.6. Immune infiltration analysis

To investigate the immune microenvironment in AF, the CIBERSORT algorithm was applied to estimate the relative proportions of 22 immune cell types in the GSE79768 dataset. Comparisons between AF and healthy control samples were performed using the Wilcoxon rank-sum test. Correlation analyses were conducted to explore relationships between immune cell populations, with Spearman correlation coefficients used to identify positive or negative associations. Furthermore, the correlation between key AF-related genes and immune cell infiltration levels was assessed, providing insights into the potential immunomodulatory roles of these genes in AF pathology.

### 2.7. ScRNA-seq and cell communication analysis

ScRNA-seq data from GSE224995 (healthy controls) and GSE261170 (AF patients) were processed using the Seurat package in R. Standard preprocessing steps, including quality control, normalization, feature selection, and dimensionality reduction, were performed. Cells were clustered using the Uniform Manifold Approximation And Projection method, and distinct cell populations were annotated based on known marker genes. The expression distribution of key genes across different cell types was examined to identify cell-type-specific functional roles.

To explore intercellular communication networks, the CellPhoneDB database was utilized to analyze ligand-receptor interactions among different cell types. Differential cell-cell communication patterns between AF and healthy samples were identified, highlighting potential signaling pathways involved in AF pathogenesis.

## 3. Results

### 3.1. Differential gene expression analysis and visualization

Differential expression analysis was conducted on the GSE79768 dataset using the “limma” package, comparing atrial tissue samples from AF patients and healthy controls. Genes with |log_2_ fold change| > 1.0 and *P* value < .05 were considered significantly differentially expressed.

**Figure 1.**
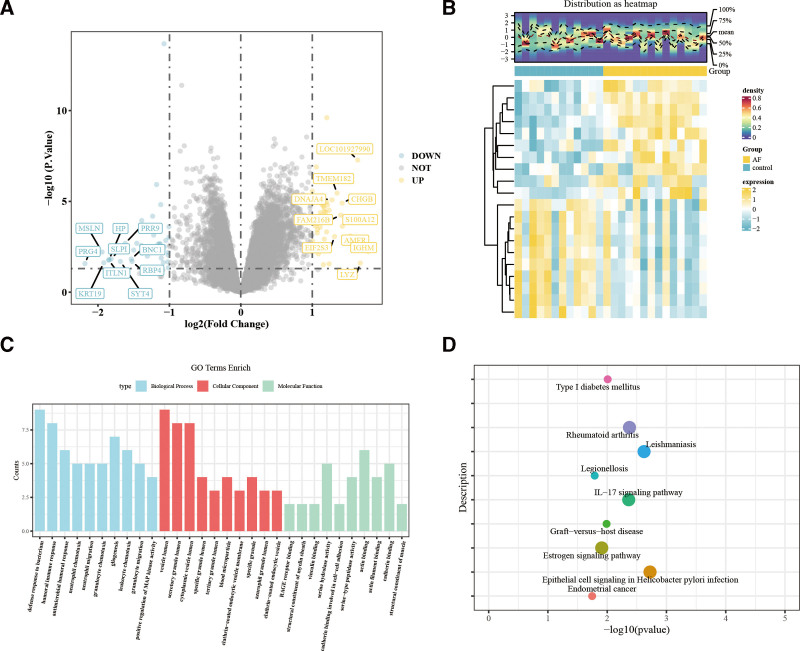
Identification of key genes and functional enrichment analysis in AF. (A) Volcano plot illustrating DEGs between the AF and control groups. The x-axis represents log_2_ (Fold Change), while the y-axis represents −log_10_ (*P* value). Blue-labeled genes indicate significantly downregulated genes (DOWN), yellow-labeled genes represent significantly upregulated genes (UP), and gray dots correspond to nonsignificant genes (NOT). (B) Heatmap depicting the expression profiles of DEGs between AF and control groups. The top density plot (Distribution as heatmap) shows the expression distribution across samples. The heatmap color scale represents Z-score normalized expression values, with blue indicating low expression and yellow indicating high expression. (C) GO enrichment analysis of DEGs, categorized into Biological Process (blue), Cellular Component (red), and Molecular Function (green). The y-axis indicates the gene count within each GO term. (D) KEGG pathway enrichment analysis highlighting significantly enriched pathways associated with AF. The x-axis represents −log_10_ (*P* value), and the y-axis lists enriched pathways. Colored dots indicate different pathway categories, with immune-related and disease-associated pathways prominently represented. AF = atrial fibrillation, DEG = differentially expressed genes, GO = Gene Ontology, KEGG = Kyoto Encyclopedia of Genes and Genomes.

A total of 85 DEGs were identified, of which 48 were upregulated and 37 downregulated. A volcano plot (Fig. 1A) was generated using “ggplot2” to visualize global gene expression changes, highlighting the top 10 upregulated and downregulated genes. A corresponding heatmap (Fig. 1B) of these top 20 genes illustrates distinct expression patterns between AF patients and controls, indicating their potential involvement in AF pathogenesis.

### 3.2. Functional enrichment analysis of DEGs

To further elucidate the potential roles of these DEGs in AF pathogenesis, GO and KEGG pathway enrichment analyses were conducted to identify associated biological processes and signaling pathways (Fig. 1C and 1D). Using the “clusterProfiler” package, GO and KEGG enrichment analyses were performed, and pathways with *P* < .05 were selected. The top 10 most significantly enriched pathways were visualized. GO analysis revealed that DEGs were predominantly enriched in immune response and inflammatory pathways, such as antimicrobial immune response and neutrophil function, suggesting a potential role of immune dysregulation in AF progression. In terms of cellular components, DEGs were significantly enriched in vesicle lumen and specific granules, indicating that vesicle-mediated secretion or signal transduction may play a critical role in AF molecular mechanisms. In molecular function analysis, genes were enriched in cell adhesion (e.g., cadherin binding) and actin cytoskeleton regulation, suggesting that AF may be associated with altered cell-cell interactions and cytoskeletal remodeling (Fig. 1C). KEGG pathway analysis further revealed that DEGs were significantly enriched in immune-inflammatory pathways such as the interleukin-17 signaling pathway and were closely associated with immune disorders such as rheumatoid arthritis and graft-versus-host disease (Fig. 1D). This suggests that AF may be accompanied by immune system dysregulation and chronic inflammatory responses.

### 3.3. Key gene selection and diagnostic value assessment

**Figure 2.**
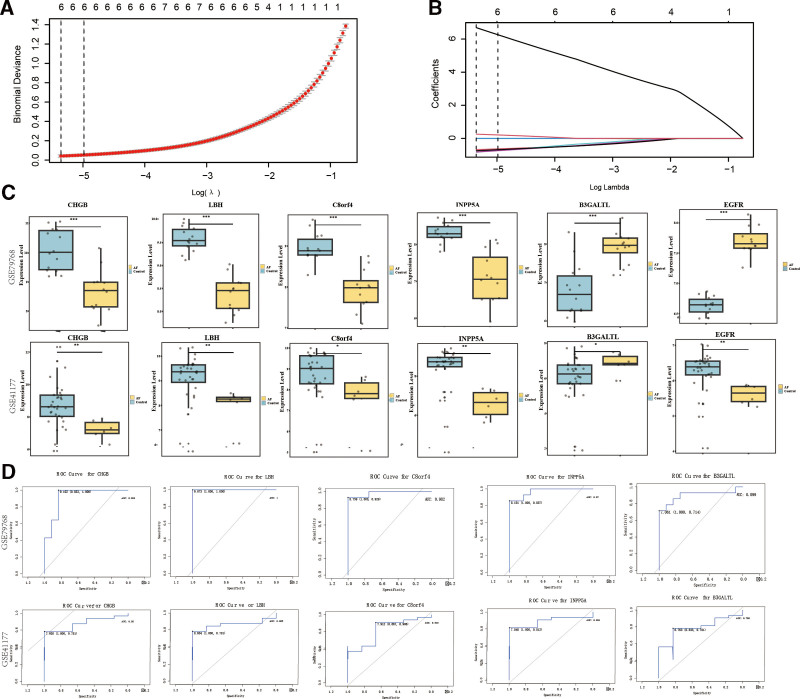
Identification and validation of key AF-related genes using LASSO regression and ROC analysis. (A) LASSO regression model was constructed to select the optimal set of AF-related genes. The y-axis represents the binomial deviance, and the x-axis represents log(λ). The vertical dashed line indicates the optimal λ value. (B) LASSO coefficient profils of candidate genes. The x-axis represents log(λ), and the y-axis represents the coefficient values. The vertical dashed line marks the optimal λ, leading to the selection of 6 key genes: CHGB, LBH, C8orf4, INPP5A, B3GALTL, and EGFR. (C) Boxplots showing differential expression of the 6 identified genes in AF patients and controls across 2 datasets (GSE79768 and GSE41177). Asterisks indicate statistical significance (*P < .05, **P < .01, ***P < .001). (D) ROC curve analysis evaluating the diagnostic performance of the selected genes in distinguishing AF patients from controls in both datasets. The AUC values demonstrate high diagnostic accuracy (AUC ≥ 0.7) for LBH, C8orf4, INPP5A, CHGB, and B3GALTL, indicating their potential as biomarkers for AF. AF = atrial fibrillation, AUC = area under the curve, LASSO = least absolute shrinkage and selection operator, ROC = receiver operating characteristic.

To identify key AF-related genes, LASSO regression analysis was conducted using the “glmnet” package, ultimately identifying 6 core genes: EGFR, LBH, C8orf4, INPP5A, CHGB, and B3GALTL (Fig. 2A and 2B). Expression analysis in the validation dataset (GSE41177) and the training dataset (GSE79768) showed that, except for EGFR, the remaining 5 genes (LBH, C8orf4, INPP5A, CHGB, and B3GALTL) exhibited consistent and significant expression trends across both datasets, indicating their robustness and potential biological relevance in AF (Fig. 2C). Additionally, ROC curve analysis was used to evaluate the diagnostic performance of these key genes, and AUC values were calculated. The results demonstrated that LBH, C8orf4, INPP5A, CHGB, and B3GALTL all achieved AUC **≥ **0.**7**, suggesting their potential as molecular biomarkers for AF diagnosis (Fig. 2D).

### 3.4. Functional analysis of key genes via GSEA

**Figure 3.**
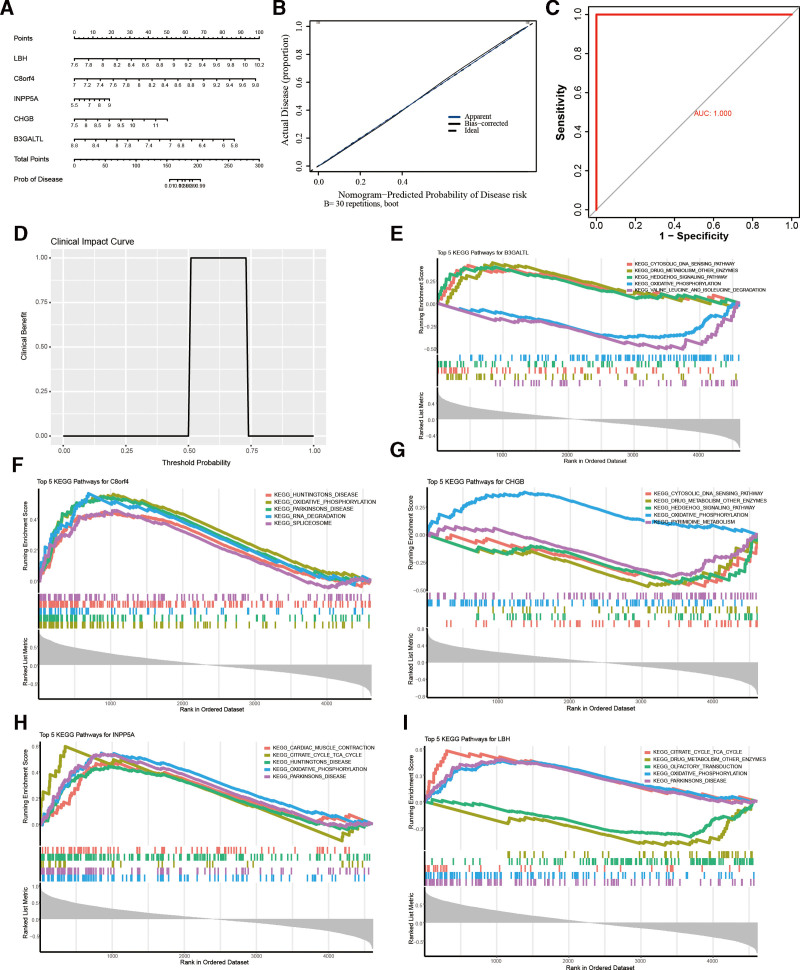
Construction and validation of the nomogram model and functional enrichment analysis of key AF-related genes. (A) Nomogram model for predicting AF risk based on the 6 key genes (LBH, C8orf4, INPP5A, CHGB, B3GALTL, and EGFR). Each gene corresponds to a score, and the total score is used to estimate the probability of AF occurrence. (B) Calibration curve assessing the predictive accuracy of the nomogram model. The closer the blue line (actual) is to the diagonal (ideal), the better the predictive performance. (C) ROC curve of the nomogram model, demonstrating high diagnostic accuracy with an AUC of 1.000. (D) Clinical impact curve showing the estimated probability of AF occurrence based on the nomogram model. (E–I) GSEA for the top 10 enriched pathways associated with B3GALTL (E), C8orf4 (F), CHGB (G), INPP5A (H), and LBH (I), highlighting their potential biological functions in AF. AF = atrial fibrillation, AUC = area under the curve, GSEA = gene set enrichment analysis, KEGG = KEGG = Kyoto Encyclopedia of Genes and Genomes, ROC = receiver operating characteristic.

A nomogram model was constructed to predict AF risk using 6 key genes: LBH, C8orf4, INPP5A, CHGB, B3GALTL, and EGFR. In this model (Fig. 3A), each gene is assigned a specific score, and the cumulative total score is employed to estimate the probability of AF occurrence. The calibration curve (Fig. 3B) was utilized to assess the predictive accuracy of the nomogram model. The proximity of the blue line (representing the actual data) to the diagonal line (the ideal scenario) indicates superior predictive performance. The ROC curve of the nomogram model (Fig. 3C) demonstrated an exceptionally high diagnostic accuracy, with an AUC of 1.000. The clinical impact curve (Fig. 3D) depicted the estimated probability of AF occurrence based on the nomogram model, providing a practical tool for clinical decision-making.

GSEA analysis revealed that C8orf4 was primarily enriched in neurodegenerative diseases (Huntington disease, Parkinson disease), RNA metabolism (RNA degradation, spliceosome), and oxidative phosphorylation pathways, indicating its potential role in AF neuroregulation and metabolic processes (Fig. 3D). CHGB was significantly associated with immune responses (cytoplasmic DNA sensing pathway), Hedgehog signaling, oxidative phosphorylation, and pyrimidine metabolism, suggesting its involvement in immune regulation and energy metabolism imbalance in AF. Additionally, HSPA8 was predominantly enriched in protein folding, autophagy, and immune-related pathways, indicating its potential role in stress response and autophagy-mediated cellular protection. RPL3 and RPL13, as ribosomal proteins, were significantly associated with protein synthesis and transcriptional regulation pathways, suggesting their role in AF-related transcriptional regulation (Fig. 3D). Notably, C8orf4 and CHGB were directly linked to oxidative phosphorylation, while HSPA8, RPL3, and RPL13 may influence energy metabolism by regulating protein homeostasis and transcriptional processes (Fig. 3E–3I). These findings highlight the crucial role of mitochondrial dysfunction in AF pathogenesis.

### 3.5. Immune cell infiltration analysis

**Figure 4.**
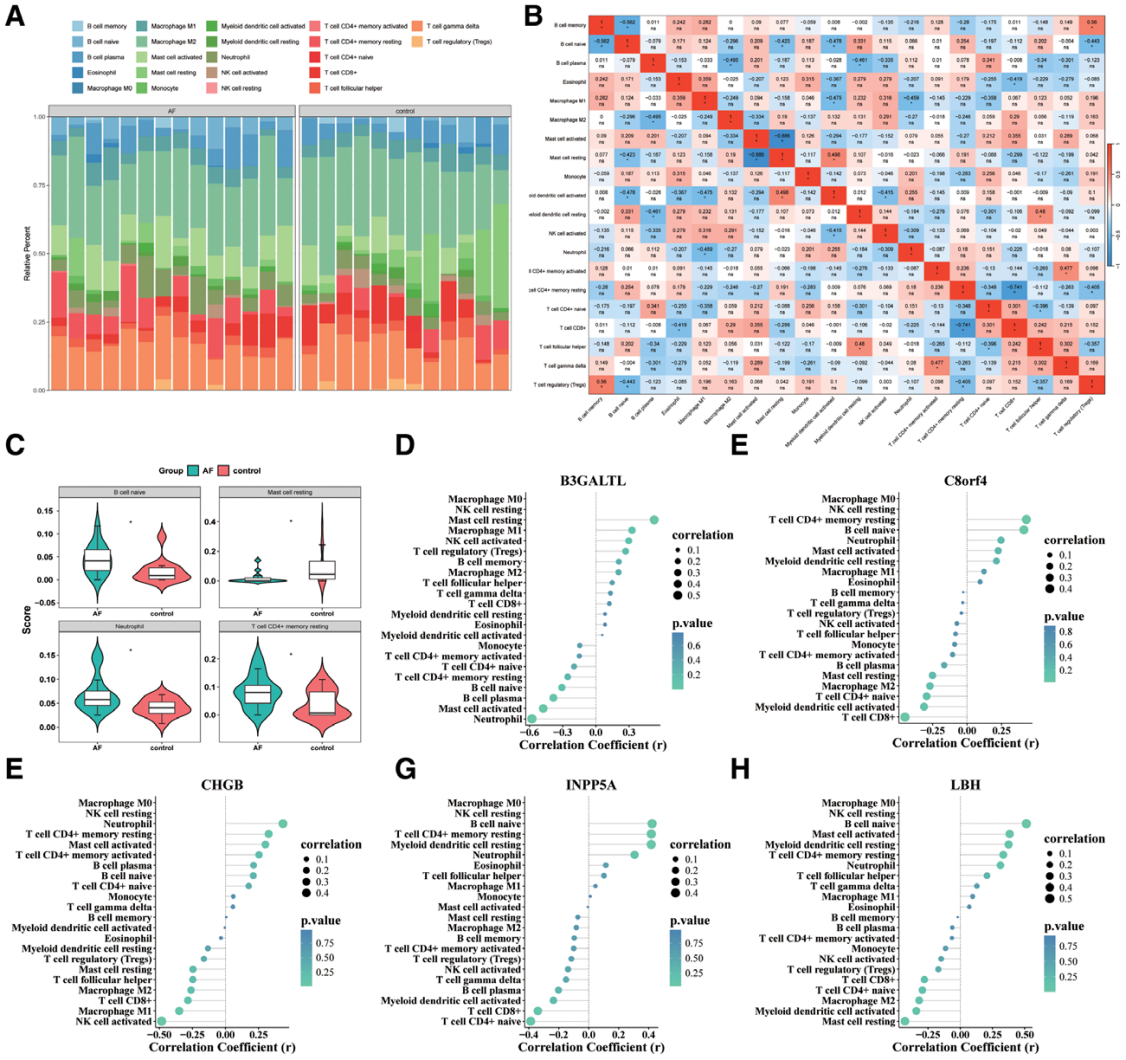
Immune cell infiltration analysis and correlation between key AF-related genes and immune cell populations. (A) Stacked bar plot showing the relative abundance of 22 immune cell types in AF and control groups, highlighting group-specific immune infiltration patterns. (B) Violin-box plots illustrating the distribution of infiltration scores for selected immune cell types (B cells naive, Mast cells resting, Neutrophils, and resting CD4^+^ memory T cells) between AF and control groups. (C) Heatmap showing the Spearman correlation matrix among immune cell types; red indicates a positive correlation, while blue indicates a negative correlation. (D–H) Spearman correlation analysis between key AF-related genes and immune cell infiltration levels: B3GALTL (D), C8orf4 (E), CHGB (F), INPP5A (G), and LBH (H). R and *P* values are provided to show the strength and significance of associations. AF = atrial fibrillation, CD4^+^ = cluster of differentiation 4 positive, R = Correlation coefficients.

We utilized the CIBERSORT algorithm on the GSE64711 dataset to assess the composition of 22 immune cell types. The analysis showed that patients with AF had a significantly higher infiltration of neutrophils, classically activated macrophages (M1 macrophages), and activated dendritic cells, while the proportions of alternatively activated macrophages (M2 macrophages), resting cluster of differentiation 4 positive (CD4^+^) memory T cells, and regulatory T cells (Tregs) were markedly reduced compared to healthy individuals (Fig. 4A). These alterations suggest an enhanced pro-inflammatory state in AF, potentially accompanied by impaired immunoregulatory mechanisms that may contribute to tissue injury and autoimmune responses.

Detailed examination of individual immune populations revealed that neutrophils and naive B cells were more abundant in AF samples, pointing to intensified inflammatory activity. In contrast, the control group showed elevated levels of resting mast cells, suggesting more effective immune modulation in non-AF individuals. Notably, resting CD4^+^ memory T cells were also increased in AF, possibly reflecting ongoing immune activation (Fig. 4B).

Spearman correlation analysis revealed robust interrelationships between immune cell subsets, such as a positive correlation between B cells and Tregs, and a negative association between M1 and M2 macrophages. These findings imply that disturbances in immune equilibrium may underlie the pathogenesis of AF (Fig. 4C).

Correlation studies involving key genes showed that LBH, C8orf4, and CHGB were positively linked with inflammatory cells, including M1 macrophages, neutrophils, and CD4^+^ memory T cells, indicating that these genes may play roles in fostering a pro-inflammatory milieu. Conversely, INPP5A and B3GALTL demonstrated positive correlations with M2 macrophages and Tregs, suggesting their involvement in anti-inflammatory responses and immune regulation (Fig. 4D–4H).

Together, the data highlight a disrupted balance between inflammatory and regulatory immune cells in AF, with specific genes potentially contributing to the modulation of atrial immune microenvironments.

### 3.6. Single-cell transcriptomic analysis

**Figure 5.**
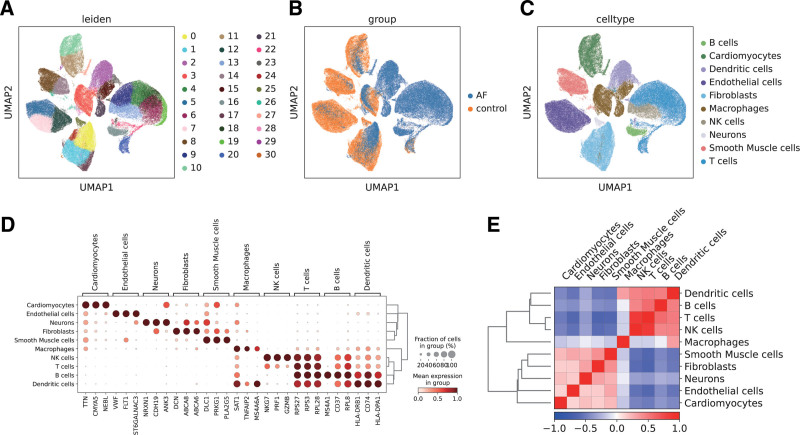
scRNA-seq analysis of AF and healthy control samples. (A) UMAP clustering of single cells colored by Leiden clusters, identifying 31 distinct cell clusters. (B) UMAP visualization of cells colored by sample group (AF patients vs healthy controls). (C) UMAP clustering of cells annotated by cell type, including cardiomyocytes, endothelial cells, smooth muscle cells, immune cells (macrophages, T cells, B cells, NK cells, dendritic cells), fibroblasts, and neurons. (D) Dot plot showing the expression of canonical marker genes across identified cell types. Dot size represents the proportion of expressing cells; color intensity reflects average expression. (E) Heatmap showing the Spearman correlation of average gene expression among major cell types. AF = atrial fibrillation, scRNA-seq = single-cell RNA sequencing, UMAP = Uniform Manifold Approximation and Projection.

ScRNA-seq data from GSE224995 (healthy controls) and GSE261170 (AF patients) underwent unsupervised clustering, which revealed 31 unique cell clusters. These were subsequently consolidated into 9 principal cell types for clearer interpretation: cardiomyocytes, endothelial cells, fibroblasts, smooth muscle cells, T cells, B cells, macrophages, neutrophils, and dendritic cells (Fig. 5A–5C). As shown in Figure 5D, a heatmap of representative marker gene expression profiles across clusters confirmed the accuracy of cell classification. To further elucidate intercellular dynamics, a correlation heatmap was generated to assess relationships among the 9 major cell types (Fig. 5E). Strong positive associations were noted between macrophages and dendritic cells, as well as smooth muscle cells and endothelial cells, indicating possible co-regulation or shared functional roles. Conversely, fibroblasts exhibited negative correlations with several immune cell populations, suggesting distinct roles in structural support versus immune response.

### 3.7. Cell-type-specific expression and intercellular communication of key genes in AF

**Figure 6.**
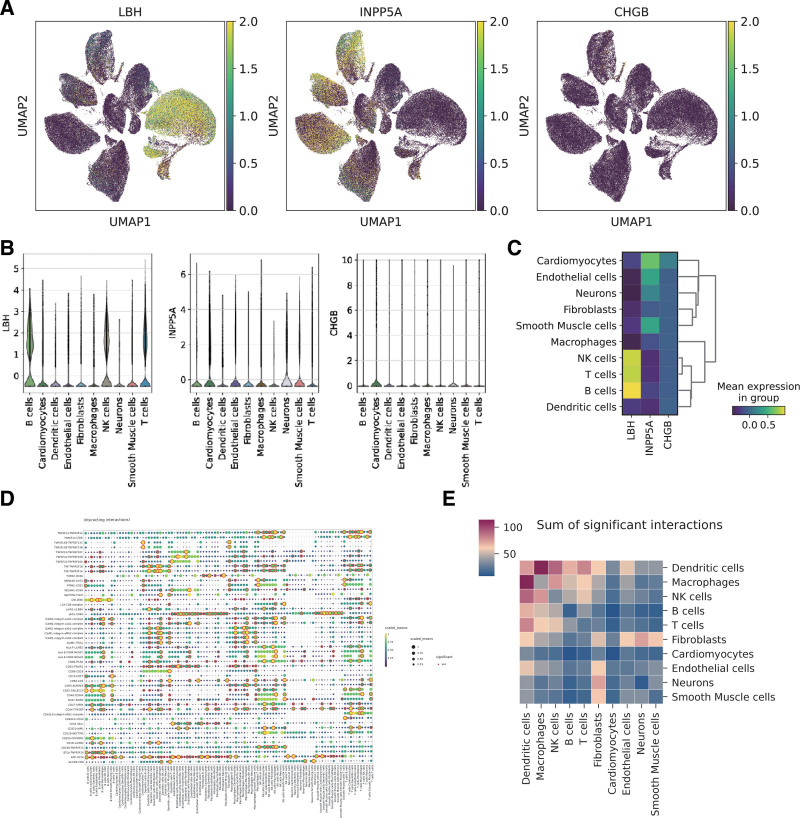
Expression and interaction analysis of key genes in AF and healthy control samples. (A) UMAP visualization of single-cell RNA sequencing data showing the expression levels of LBH, INPP5A, and CHGB across different cell types. (B) Violin plots depicting the expression distribution of LBH, INPP5A, and CHGB in various

To further elucidate the cellular context of key AF-related genes, we analyzed their expression patterns using scRNA-seq data. Uniform Manifold Approximation And Projection plots (Fig. 6A) demonstrated that LBH, INPP5A, and CHGB exhibit distinct spatial expression profiles across various cell types. Specifically, LBH was primarily expressed in B cells, INPP5A was enriched in T cells and neutrophils, while CHGB showed limited expression, suggesting a highly cell-specific role. Violin plots (Fig. 6B) and an expression heatmap (Fig. 6C) confirmed these cell-type-specific patterns, highlighting potential functional relevance of these genes in immune regulation and tissue remodeling.

cell types, highlighting their cell type-specific expression patterns. (C) Heatmap showing the mean expression of LBH, INPP5A, and CHGB across different cell

types, with hierarchical clustering indicating similarities in expression profiles. (D) Dot plot depicting selected ligand-receptor pairs involved in intercellular communication between different cell types. Dot size reflects the interaction strength, and color intensity represents the average expression level of each ligand-receptor pair. (E) Heatmap summarizing the overall intercellular communication strength (based on all significant ligand-receptor pairs) between major cell types. AF = atrial fibrillation, UMAP = Uniform Manifold Approximation and Projection.

Next, ligand-receptor interaction analysis was conducted to explore intercellular communication in AF. The bubble chart (Fig. 6D) illustrated key ligand-receptor pairs mediating interactions between immune and stromal cells, such as macrophage (endothelial and T cell) fibroblast signaling axes. (Fig. 6E)

## 4. Discussion

AF is a complex and heterogeneous disorder involving structural, electrical, and metabolic remodeling.^[13–15]^ Our study provides a comprehensive molecular analysis of AF using multiple transcriptomic datasets, identifying key genes, immune infiltration patterns, and potential therapeutic targets.

One of the most notable findings of this study is the identification of immune dysregulation as a key factor in AF pathogenesis. Our analysis revealed increased levels of pro-inflammatory immune cells (e.g., neutrophils, M1 macrophages, and activated dendritic cells) and a reduction in anti-inflammatory cells (e.g., M2 macrophages, Tregs, and resting CD4^+^ memory T cells). These findings suggest that AF is associated with a chronic inflammatory state, which may contribute to atrial fibrosis and electrical remodeling. Prior studies have also reported elevated inflammatory cytokines, such as IL-6 and tumor necrosis factor-alpha, in AF patients, supporting our hypothesis that immune dysregulation plays a central role in disease progression.^[16,24]^ However, most existing studies focus on systemic inflammation markers, while our study provides a more detailed cell-type-specific immune profiling, adding novel insights into AF-related immune mechanisms.

In addition to immune dysfunction, our results highlight the role of oxidative phosphorylation and mitochondrial dysfunction in AF pathogenesis. GSEA revealed that key genes such as C8orf4 and CHGB were significantly enriched in oxidative phosphorylation and energy metabolism pathways. Given that oxidative stress has been widely implicated in AF development, our findings further emphasize the importance of metabolic dysfunction in the disease. Previous studies have demonstrated increased mitochondrial reactive oxygen species in AF patients, which can contribute to atrial remodeling.^[25,26]^ The association between key AF genes and mitochondrial function provides a new perspective on how metabolic dysregulation may contribute to AF onset and progression.

Another key discovery in our study is the identification of LBH, C8orf4, INPP5A, CHGB, and B3GALTL as potential AF biomarkers and therapeutic targets. These genes were consistently differentially expressed across multiple datasets and exhibited strong diagnostic value (AUC ≥ 0.7). While our study provides important insights into the molecular mechanisms of AF, there are some limitations that should be acknowledged. First, our findings are primarily based on bioinformatics analysis of publicly available datasets, which, while powerful, require experimental validation. Functional studies, such as knockout/overexpression experiments in cell or animal models, are needed to confirm the roles of these key genes in AF pathogenesis. Additionally, differences in patient demographics, comorbidities, and medication use across datasets may introduce variability in gene expression patterns, highlighting the need for prospective cohort studies to validate these biomarkers in diverse patient populations.

## Acknowledgements

We sincerely thank all data contributors who provided access to public datasets in the GEO database. We also thank all members of our research group at The First Affiliated Hospital of Fuzhou for their technical support and helpful discussions.

## Author contributions

**Conceptualization:** Xiaolan Lin, Yan Zhang.

**Data curation:** Chongkun Zheng, Laiying Xu, Yan Zhang.

**Formal analysis:** Zhijie Ye, Yan Zhang.

**Funding acquisition:** Yilin Su, Yan Zhang.

**Investigation:** Laiying Xu, Yan Zhang.

**Methodology:** Yilin Su, Junliang Hou, Yan Zhang.

**Project administration:** Yilin Su.

**Resources:** Yan Zhang.

**Supervision:** Yilin Su, Xiaolan Lin.

**Validation:** Zhijie Ye, Junliang Hou, Yan Zhang.

**Visualization:** Chongkun Zheng.

**Writing – original draft:** Yan Zhang.

**Writing – review & editing:** Yilin Su.
